# An Ionizing Radiation Sensor Using a Pre-Programmed MAHAOS Device

**DOI:** 10.3390/s140814553

**Published:** 2014-08-11

**Authors:** Wen-Ching. Hsieh, Hao-Tien Daniel. Lee, Fuh-Cheng. Jong

**Affiliations:** 1 Department of Opto-Electronic System Engineering, Minghsin University of Science and Technology, Xinxing Rd, 1, Xinfeng 30401, Taiwan; 2 ETOMS Electronics Corp, 12, Innovation 1st. Rd, Science-Based Industrial Park, Hsin-Chu 300, Taiwan; E-Mail: danieleewww@gmail.com; 3 Electronic Engineering Department, Southern Taiwan University of Science and Technology, 1, Nan-Tai Street, Yungkang District, Tainan 710, Taiwan; E-Mail: fcjong@mail.stust.edu.tw

**Keywords:** high *k*, sensor, radiation, SONOS, SOHOS, MOS

## Abstract

Metal-aluminum oxide–hafnium aluminum oxide–silicon oxide–silicon (hereafter MAHAOS) devices can be candidates for ionizing radiation sensor applications. In this work, MAHAOS devices (SONOS-like structures with high *k* stack gate dielectric) were studied regarding the first known characterization of the ionization radiation sensing response. The change of threshold voltage V*_T_* for a MAHAOS device after gamma ray exposure had a strong correlation to the total ionization dose (TID) of gamma radiation up to at least 5 Mrad TID. In this paper, the gamma radiation response performances of the pre-programmed and virgin (non-pre-programmed) MAHAOS devices are presented. The experimental data show that the change of V*_T_* for the pre-programmed MAHAOS device with gamma irradiation is very significant. The data of pre-programmed MAHAOS devices written by 5 Mrad TID of gamma radiation was also stable for a long time with data storage. The sensing of gamma radiation by pre-programmed MAHAOS devices with high *k* stack gate dielectric reported in this study has demonstrated their potential application for non-volatile ionizing radiation sensing technology in the future.

## 1. Introduction

**Figure 1.**
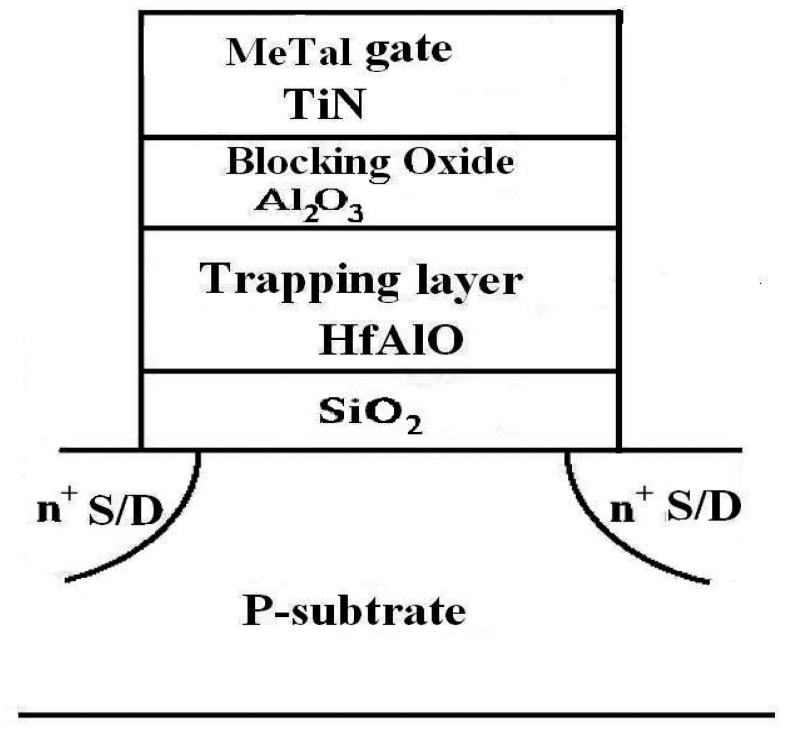
Schematic cross-section of MAHAOS capacitor devices in this work.

After gamma irradiation, it is well known that the MOS structure has a build-up of positive charges at the Si-SiO_2_ interface and an interface state occurs in the structure [1]. The radiation effects of a Metal-Nitride-Oxide-Silicon (MNOS) device with stacked insulation layers composed of silicon nitride and silicon dioxide has been reported [2–6]. Total ionization dose (TID) radiation effects in traditional silicon-oxide-nitride-oxide-silicon (SONOS) non-volatile memory (NVM) devices have been studied previously [7–9]. Until now, little was known about the radiation response of SONOS–like devices with high *k* gate dielectric structure. The poly–silicon oxide-high *k* oxide-silicon oxide-silicon (so-called SOHOS) structure with hafnium oxide (HfO_2_) for charge storage layers demonstrates superior charge storage capability at low voltages, faster programming and less over-erase problems compared to conventional SONOS NVMs, but it has poorer charge retention capability. The SOHOS with hafnium aluminum oxide HfAl_x_O_y_ (hereafter HfAlO) for charge storage layer previously showed advantages of high speed program/erase and good charge-retention capability, which renders SOHOS with HfAlO charge storage the most promising candidate for NVM device application [10–13]. Until now, little was known about the radiation sensing response performance of metal-aluminum oxide hafnium aluminum oxide–silicon oxide–silicon (hereafter MAHAOS) devices. In this work, we built a MAHAOS device and performed the first known characterization of the total ionization dose (TID) sensor response on these devices. This new type of MAHAOS device is illustrated in Figure 1. The gate dielectric stack of this MAHAOS device is made of SiO_2_, HfAlO and Al_2_O_3_. In this paper, the radiation response performances of MAHAOS devices with pre-programmed and virgin (non-pre-programmed) state were characterized.

**Figure 2.**
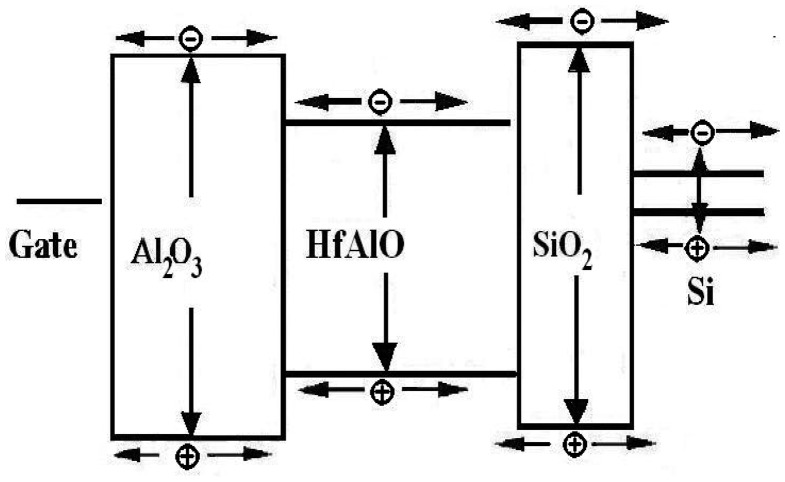
Charge generation and trapping for the MAHAOS during irradiation.

Figure 2 shows a graphical view of charge generation and trapping states of the gate dielectric for the MAHAOS device during gamma irradiation. In an ionizing radiation environment, hole trapping is usually the dominant source of radiation-induced oxide-trap charge in SONOS-type devices [8]. The MAHAOS devices using high *k* gate dielectric materials, which also can be integrated into the mainstream CMOS semiconductor processes, were studied and in this paper the first known characterization of the TID sensing response by applying ionizing radiation is reported.

## 2. Experimental Details

**Figure 3.**
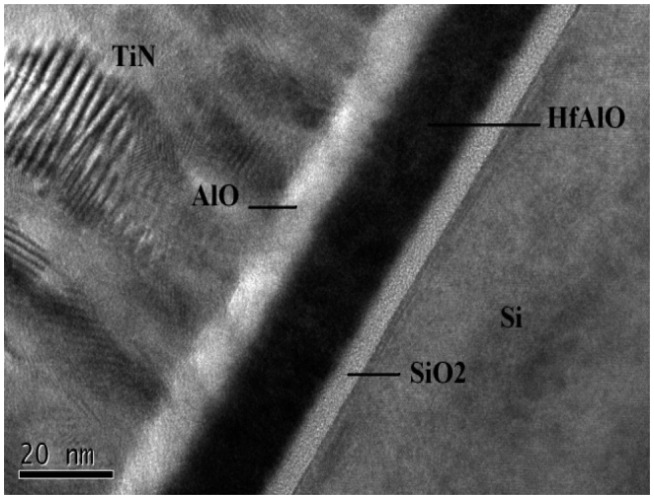
Transmission electron micrograph showing details of the MAHAOS gate stack region.

The *N*-channel MAHAOS device were fabricated at the Taiwan National Nano Device Laboratories (NDL, Hsin-Chu, Taiwan). Starting wafers were 6” silicon (100) orientation with boron doped type substrates with a resistivity of 15–25 Ω·cm. A tunneling SiO_2_ was formed by an advanced clustered vertical furnace on the cleaned wafers and its thickness was 4 ∼ 6 nm, which was measured by spectroscopic ellipsometry. After the tunneling oxide formation, HfAlO films were deposited as the charge-trapping layers in a metal organic chemical vapor deposition (MOCVD) system at 400 ∼ 550 °C with 10 ∼ 20 nm thick deposition. A composition ratio between 20% and 30% of Al in HfAlO is achieved by controlling the deposition process parameters. HfAlO films were deposited by using two precursors, Hf (tert-butoxy)_2_(mmp)_2_ and aluminum isopropoxide in the MOCVD system. The top oxide Al_2_O_3_ was formed by MOCVD at 450 ∼ 550 °C to an approximate thickness of 5 ∼ 10 nm thick, and a 200 ∼ 400 nm TiN metal gate was formed by the DC sputtering for the control gate. The resulting structure is illustrated the Figure 1. After gate patterning, source and drain were formed by implantation with arsenic atoms which were activated at 900 °C for 30 s. A TEM image of the resulting MAHAOS gate stack structure is illustrated in the Figure 3.

The basic wafer-level program-erase testing was performed at room temperature by using a HP4156A parameter analyzer together with an Agilent HP16440A pulse generator and a cascade probe station before gamma radiation. In this paper, the radiation response performances of MAHAOS devices with pre-programmed and virgin states were characterized and compared. Before irradiation, these samples were pre-programmed by apply a positive gate voltage first and then were irradiated with zero gate bias (also zero source-drain bias) for TID test. This MAHAOS device is pre-programmed by using F-N tunneling of electrons from the substrate into the HfAlO trapping layer with positive gate voltage prior to gamma irradiation. The gamma radiation was performed on the MAHAOS by using a ^60^Co irradiator at room temperature. Gate leakage current *vs.* gate voltage (I*_G_*-V*_G_*) curves were obtained by using a computer-controlled HP4156 parameter analyzer and gate capacitance *vs.* gate voltage (C*_G_*-V*_G_*) curve measurements were obtained by using a computer-controlled HP4284 parameter analyzer. The C*_G_*-V*_G_* curve is measured by sweeping gate voltage together with zero source and drain bias conditions. Pre-irradiation and post-irradiation, I*_G_*-V*_G_* and C*_G_*-V*_G_* curve behaviors of this MAHAOS device are also compared. Radiation-induced V*_T_* shifts and gate leakage current were estimated from I*_G_*-V*_G_* and C*_G_*-V*_G_* curve measurements at room temperature.

## 3. Results and Discussion

### 3.1. Capacitance-Voltage Measurements

**Figure 4.**
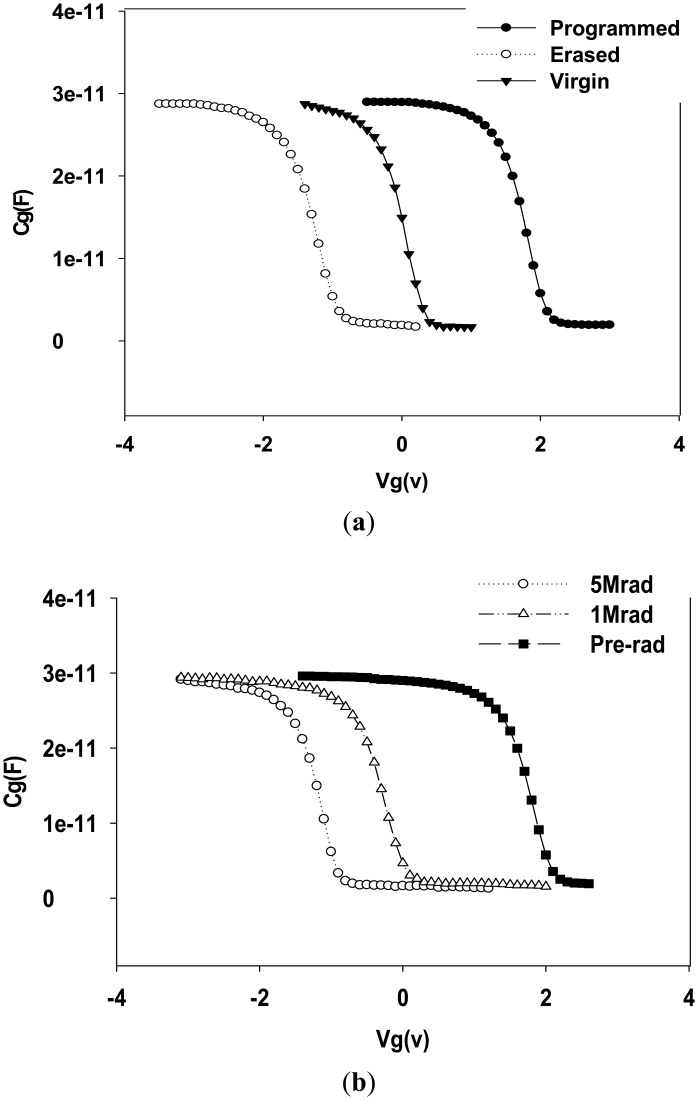
(**a**) Pre-irradiation C-V characteristic of a single MAHAOS device; (**b**) C-V curves for pre-programmed MAHAOS device with radiation up to 5 Mrad TID.

Figure 4a shows the pre-irradiation C-V characteristics for a typical MAHAOS device in the programmed and erased states. The gate capacity of this C-V curve is measured by sweeping gate voltage together with zero source and drain bias. The MAHAOS device was programmed by F-N tunneling of electrons from the substrate into the trapping dielectric using a positive gate voltage, the electrons are thus trapped in the trapping layer and there is a large positive shift in V*_T_* by applying a negative gate voltage. Similarly, the MAHAOS device was erased with a negative voltage applied to the gate, holes are injected through the oxide and into the trapping layer; therefore, the holes are trapped in the HfAlO, and there is a large negative shift in V*_T_*. The magnitude of the initial V*_T_* is dependent on the frequency, duration, and amplitude of the write/erase pulses. The center curve of Figure 4a represents the initial C-V condition of a MAHAOS device and it is so called “virgin state” in this paper, which has never been written or erased previously. The initial V*_T_* of virgin state MAHAOS depends on the trapped charges located at the defects and interfaces in the gate dielectric with aluminum oxide–hafnium aluminum oxide–silicon oxide (hereafter A-HA-O) stack during wafer fabrication process before irradiation. The packet of trapped electrons due to programming and erasure changes the threshold voltage of the transistor, and causes a shift in the C-V curves. The V*_T_* of the programmed MAHAOS deice is shifted by about 3 V regardless of the magnitude of its V*_T_* in the erased state prior to gamma radiation.

Figure 4b shows a family of C-V curves for a typical single pre-programmed MAHAOS device which was irradiated stepwise up to 5 Mrad TID. A significant negative shift is observed for the pre-programmed MAHAOS device as a result of a large loss of charges in the trapping layer caused by the ionizing radiation. These results indicate the amount of trapped electron is smaller than the amount of trapped hole in the gate dielectric.

The net positive charges are trapped in the gate dielectric stack when the transistors are irradiated which resulting in a negative shift in the C-V curves as shown in the Figure 4b. A significant radiation-induced shift of C-V curve in the MAHAOS device is likely due to a combination of two different types of mechanism that occur. The first one is due to a loss of stored charge in the A-HA-O dielectric stack causing a V*_T_* shift. The gamma radiation causes the charges trapped in the original HfAlO trapping layer to be emitted from the traps. This loss of stored charge then causes a shift in V*_T_* The second is due to asymmetrical trapping causing a V*_T_* shift. The radiation generates electron/hole pairs in the HfAlO trapping layer which is more likely to trap holes than electrons, so this leads to a buildup of positive charge in the HfAlO trapping layer post gamma radiation. The hole charges in the gate oxide are the dominant radiation-induced charges of these devices under the gamma radiation conditions, which can be illustrated by a decrease in V*_T_* as seen in the Figure 4b. These results are in agreement with the previous results of radiation effects on SONOS studies [8,9]. As shown in Figure 4b, the V*_T_* of the pre-programmed MAHAOS device is decreased by about 3 V at 5 Mrad TID of gamma radiation, regardless of the magnitude of its V*_T_* in pre-programmed state prior to gamma radiation.

### 3.2. Threshold Voltage vs. TID Measurements

**Figure 5.**
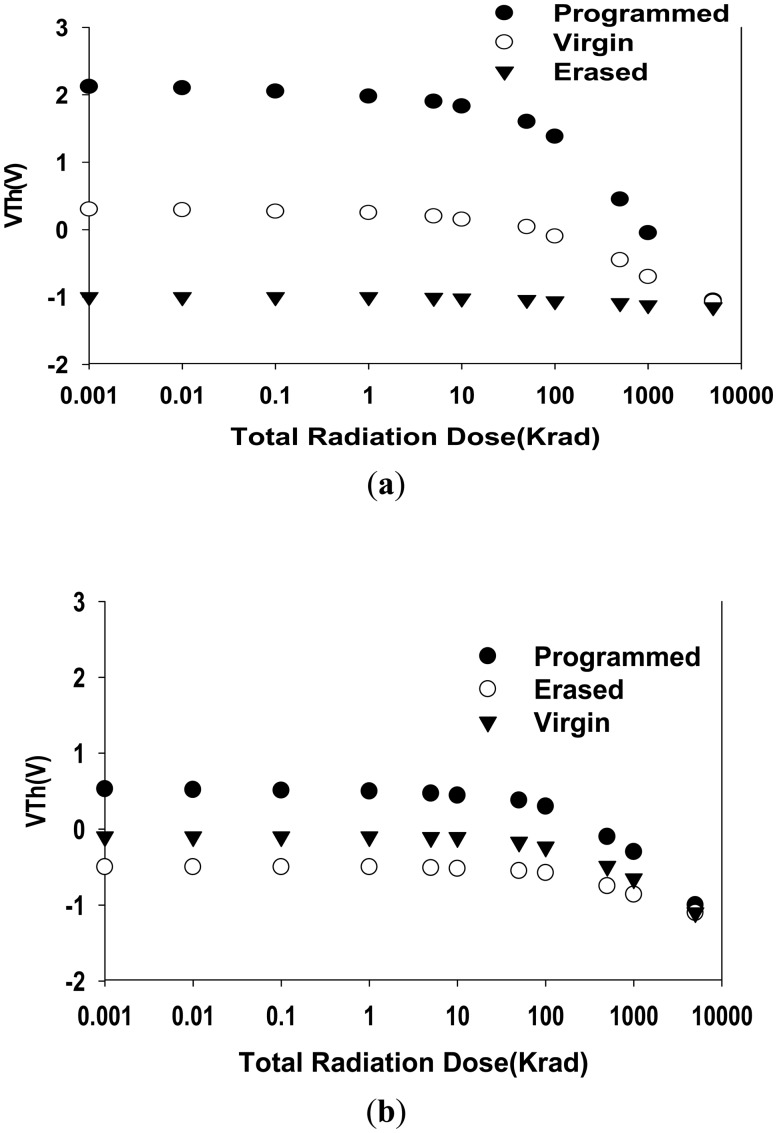

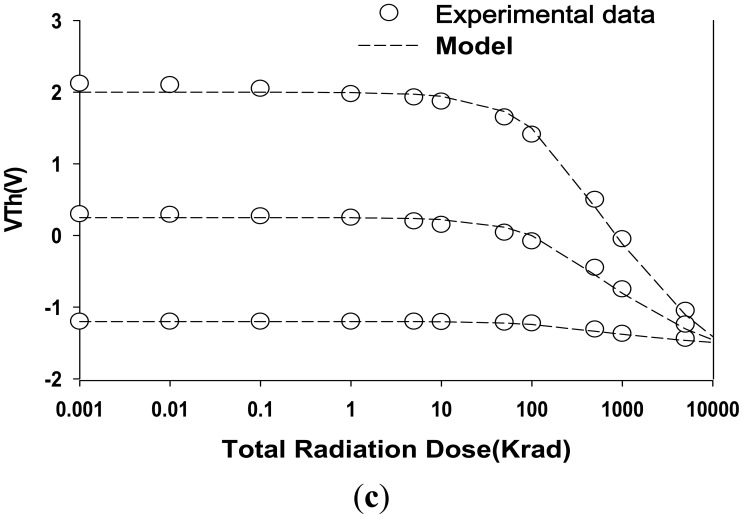
(**a**) The changes of V*_T_* decrease as a function of gamma radiation TID for MAHAOS device; (**b**) The changes of V*_T_* decrease as a function of gamma radiation TID for MONOS device; (**c**) The model of V*_T_* as a function of gamma radiation TID for MAHAOS device.

Figure 5a, further shows the change of V*_T_* as a function of gamma radiation TID for both pre-programmed and virgin (non-pre-programmed) MAHAOS devices before irradiation. The V*_T_* of the MAHAOS device in the pre-programmed, virgin, and erased states all shifted negatively after TID gamma irradiation. As the total radiation dosage increases, the difference between the threshold voltages in the programmed and the virgin states decreases. It is noted that the changes of V*_T_* does not vary significantly with stepwise irradiation up to 1 krad TID and V*_T_* decreases significantly after 1 krad TID; but V*_T_* decreases more dramatically after irradiation levels up to 100 krad TID. As shown in Figure 5a, the radiation-induced V*_T_* shifts are somewhat more significant in the irradiated and pre-programmed device compared to the irradiated and virgin device. It is clearly observed in Figure 5a that the effect of radiation on the V*_T_* of the MAHAOS device depends on the initial magnitude of V*_T_* and hence the charge trapped in the HfAlO trapping layer prior to irradiation. The experimental data show that the change of V*_T_* for the pre-programmed MAHAOS device with gamma irradiation is very prominent. The decrease of V*_T_* for the pre-programmed MAHAOS device is about 3 V after 5 Mrad TID gamma ray irradiation regardless of the magnitude of its V*_T_* value in pre-programmed state prior to gamma radiation. These radiation-induced shifts in the irradiated device are a combination of two effects; the first effect is a result from the loss of stored charge in the HfAlO trapping layer and the second effect is due to a build-up of positive charge resulted from asymmetric trapping of electrons and holes in the HfAlO trapping layer. These two combined effects cause a large shift in the programmed state but create a smaller shift in the erased state. It is noted that the MAHAOS device in the virgin state contains less stored charge to be removed upon irradiation. An erased device is one whose stored charges have been erased and in its HfAlO trapping layer contains only a small amount of stored charge to begin with. The V*_T_* shift upon gamma radiation of this single erased device is mostly due to the second effect described earlier and it is mainly contributed by the asymmetric trapping of charges in the trapping layer.

For comparison, a TiN-silicon oxide-nitride-silicon oxide-silicon device (hereafter MONOS) which using the same gate dielectric thickness and the same pre-program voltage of the MAHAOS device, as stated in Figure 5a, is also irradiated with gamma radiation up to 5 Mrad TID. Figure 5b illustrates changes of V*_T_* as a function of gamma radiation TID for the MONOS device. It is observed in Figure 5b that the effect of radiation on V*_T_* for the MONOS shows a similar trend as for the MAHAOS device. The changes of V*_T_* does not vary significantly with stepwise irradiation up to 1 krad TID and V*_T_* decreases significantly after 1 krad TID but V*_T_* decreases more dramatically after gamma irradiation levels up to 100 krad TID. Moreover, the effect of radiation on V*_T_* for the MONOS device also depends on the initial V*_T_* value of the MONOS device, but V*_T_* of the pre-programmed MONOS device shifts only about −1.5 V at 5 Mrad TID, regardless of the magnitude of its pre-programmed and pre-irradiation value. It is noted that the gamma irradiation causes a large decay of V*_T_* for the MAHAOS device, while it causes less of a shift of V*_T_* in the MONOS device. It is suggested that the MAHAOS device pre-programmed by the same gate voltage contains more stored charge to be removed upon irradiation than that for MONOS. The trap density of the HfAlO trapping layer can be increased by increasing the doping of Al atoms into HfO_2_, which further results in a high trap density generated by doping suitable Al content into the HfAlO charge-trapping layer [12,13]. Gamma radiation sensing by the pre-programmed MAHAOS devices using high *k* gate dielectric technology has demonstrated the promising potential to be used for ionizing radiation sensing application in the future.

### 3.3. Quantitative Model

A model (hereafter called HWC mode) is now presented which can be used to model the V*_T_* shift of MAHAOS device under irradiation conditions. The HWC model is derived from the prior studies of McWhorter [6] and Fengying Qiao [9], which is given by:

(1) $$V_{T}(D)=[V_{T}(0)-A]\;\{[B]\exp(-t_{n}D)+[1-B]\exp(-t_{p}D)\}+A$$

where *D* represents the TID. *t_n_* and *t_p_* are defined as the sum of emission and capture constant of electrons and holes, respectively. *A* is the constant for specific device. *B* is the combination ratio of electrons and holes effect. The unknown four parameters *t_n_*, *t_p_*, *B* and *A* for the MAHAOS device can be derived from the curve fitting of experimental data. The analysis in this study showed *t_n_* = 3E−3/Krad, *t_p_* = 3E−4/Krad, *A* = −1.5, *B* = 0.5 are the optimum fitting parameters to predict the radiation response of the MAHAOS device in this study. Once these parameters were determined, the theoretical post irradiated *V_T_* “*V_T_*(*D*)” with any prior irradiated *V_T_* “*V_T_*(0)” can be predicted by Equation (1). Figure 5c shows a graphical view of the HWC model results plotted against the experimental *V_T_*(*D*) with different *V_T_*(0) at pre-programmed, pre-erase and virgin states of MAHAOS devices, where the HWC model can fit well with the experimental data as illustrated.

### 3.4. Threshold Voltage vs. Retention Time Measurements

**Figure 6.**
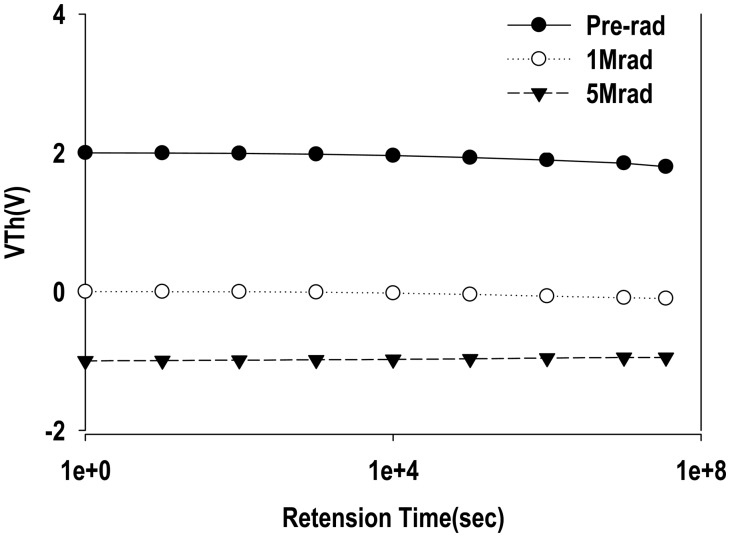
The V*_T_* stability for pre-programmed MAHAOS device after gamma irradiation.

Figure 6 shows the V*_T_* stability *vs.* retention time for the MAHAOS device after the gamma irradiation process. The upper curve is the programmed MAHAOS device before gamma irradiation and the lower curve represents the pre-programmed MAHAOS device under 1 Mrad and 5 Mrad TID gamma irradiations, respectively. It is noted that the natural decay of the threshold voltages with time for the programmed MAHAOS device prior to irradiation is a result of charge tunneling out from the HfAlO trapping layer. But the radiation-induced V*_T_* shift is very effectively recorded inside the stack gate of MAHAOS device because these MAHAOS devices with high k dielectrics are designed to trap and store charge efficiently in the gate stack. The MAHAOS device with HfAlO trapping layer takes the advantages of Al_2_O_3_ for better charge retention capability compared to SONOS [12]. As illustrated in Figure 6, after 1 Mrad TID of gamma irradiation, the V*_T_* decay rate (volts/Log(time)) of the pre-programmed MAHAOS device is not significant, even after one year at constant room temperature storage. It also shows that the decay of the V*_T_* in the pre-programmed MAHAOS device after 5 Mrad TID of gamma irradiation is not significant, but the V*_T_* did recover 0.1 V from one-year-storage after 5 Mrad TID of gamma radiation in this study. This recovery may be attributed to the partial re-programming of the memory element by the charge redistribution during a long-term room temperature annealing. These results suggest that pre-programmed MAHAOS devices would be able to sustain data for a long period under high dosage of gamma irradiation conditions.

### 3.5. Gate Leakage Current vs. Gate Voltage Measurements

**Figure 7.**
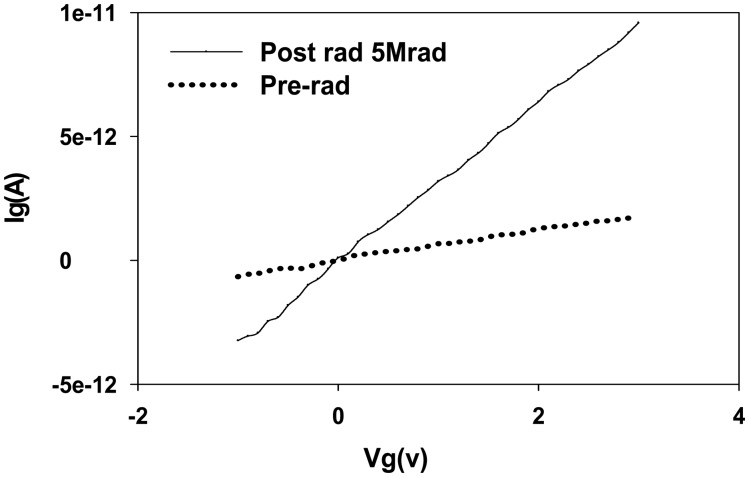
The I*_G_ vs.* V*_G_* characteristics for pre-programmed MAHAOS device post irradiation.

In Figure 7, the gate leakage current (I*_G_*) *vs.* gate voltage (V*_G_*) characteristics measured for pre-programmed MAHAOS device after 5 Mrad TID of gamma irradiation are illustrated.

The gate leakage current *vs.* gate voltage (I*_G_*-V*_G_*) curves before irradiation and after 5 Mrad TID irradiation were measured, respectively. As shown in Figure 7, the gate leakage current measured by applying 3 V gate voltage was below 0.1 pA before irradiation and was increased to 10 pA after 5 Mrad TID gamma radiation exposure. It shows the gate leakage current does not change significantly after 5 Mrad TID gamma irradiation for pre-programmed MAHAOS.

### 3.6. Gate Leakage Current vs. TID Measurements

**Figure 8.**
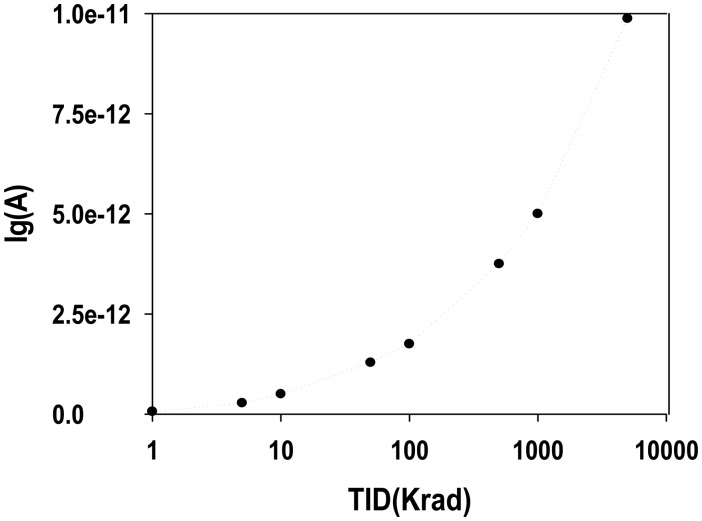
The I_G_ measured at 3 V V*_G_* for pre-programmed MAHAOS device irradiated by 5 Mrad TID.

Figure 8 illustrates the gate leakage current measured at 3 V V*_G_* for a pre-programmed MAHAOS device with gamma irradiation up to the 5 Mrad TID. The gate leakage current changes slowly before 500 krad TID gamma irradiation and changes significantly after 500 krad; but it changes more dramatically after 1 Mrad. As shown in Figure 8, the gate leakage current measured at 3 V V*_G_* was below 0.1 pA before irradiation and was increased to 10 pA after 5 Mrad. Therefore, gate leakage current is not assumed to be the main factor to contribute the V*_T_* shift of the pre-programmed MAHAOS device after gamma irradiation.

### 3.7. Gate Leakage Current vs. Retention Time Measurements

**Figure 9.**
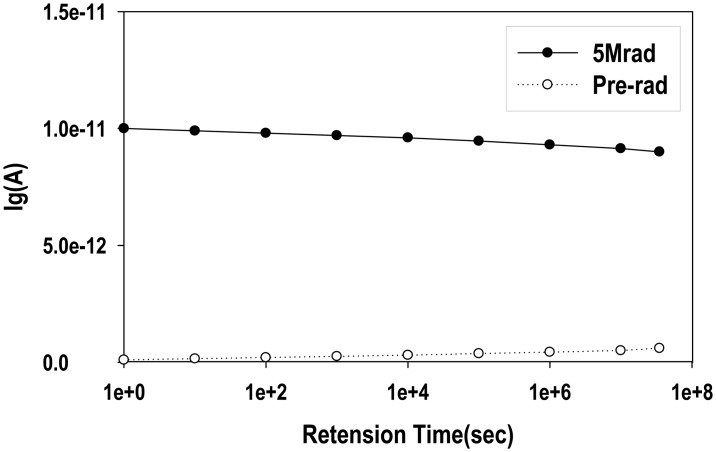
The recover of I_G_ for the pre-programmed MAHAOS device irradiated by 5 Mrad TID.

Figure 9 shows the recovery of gate leakage current *vs.* retention time for the pre-programmed and irradiated MAHAOS devices measured under 3 V V*_G_* conditions. It is noted that this gate leakage current of MAHAOS irradiated by 5 Mrad TID gamma irradiation is partially recovered by adopting one-year storage at the room temperature conditions. This effect is similar to the case of CMOS devices whose gate leakage can be partially recovered by a long-term room temperature annealing or short-term baking at 250 °C [8]. The gate leakage of pre-programmed MAHAOS devices can be annealed by applying long-term room temperature or short-term high temperature annealing as well in this study. The irreversible high dose radiation damage of programmed MAHAOS device is partially recovered in this study by applying one year storage at room temperature annealing.

### 3.8. Threshold Voltage vs. Refresh/Radiation Cycles Measurements

**Figure 10.**
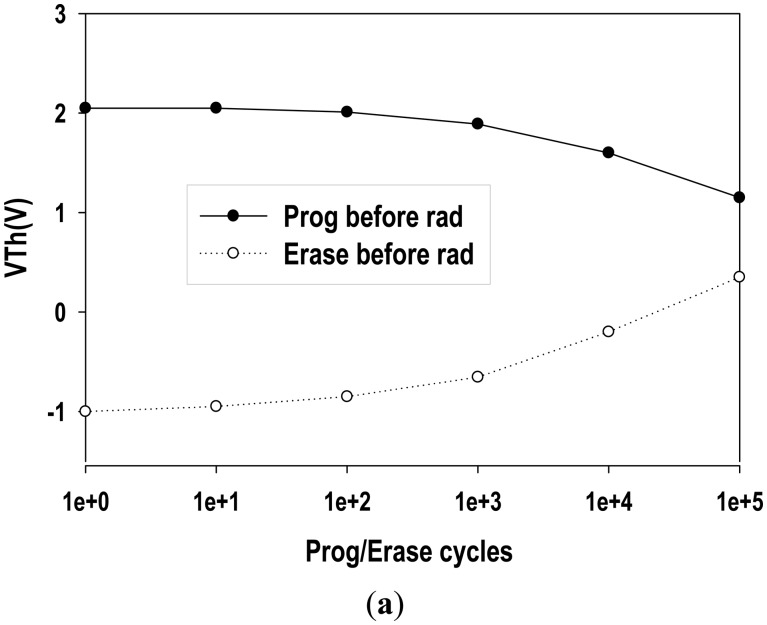

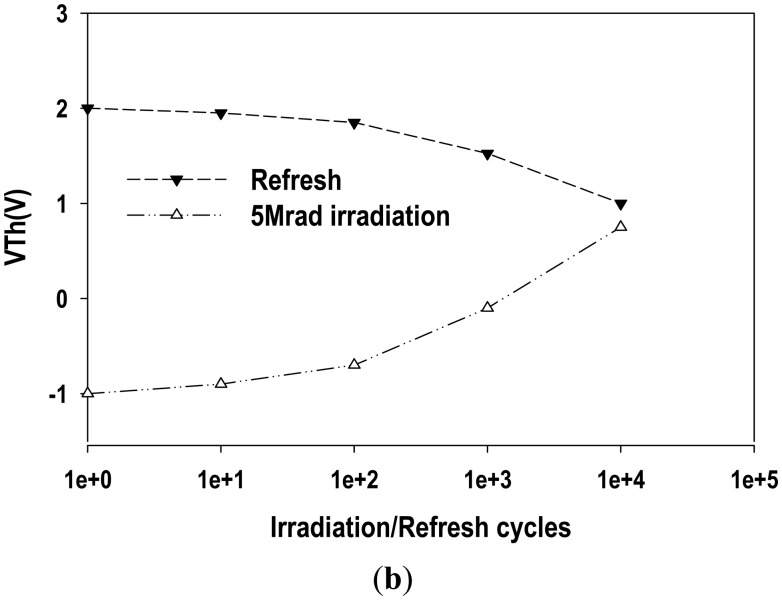
(**a**) Endurance characteristics of the MAHAOS sensor before gamma irradiation; (**b**) The 5 Mrad-irradiation/refresh cycle characteristics of the MAHAOS sensor.

Figure 10a shows the endurance characteristics of the MAHAOS device before gamma irradiation. The upper curve is the programmed MAHAOS device before gamma irradiation and the lower curve represents the erased MAHAOS device before gamma irradiation. It is noted that the V*_T_* is quite stable without change for 100 endurance cycles for MAHAOS device. As shown in the figure, the change of V*_T_* is not significant before 1000 endurance cycles and its change becomes significant after 1000 endurance cycles. It also shows that the MAHAOS device is still functional even after 100,000 endurance cycles before radiation.

Figure 10b illustrates the irradiation/refresh cycle characteristics of the MAHAOS device under high dosage of gamma irradiation conditions. There are two steps in each irradiation/refresh cycle; the first step is to expose the pre-programmed MAHAOS device under 5 Mrad TID gamma irradiation (irradiation step) and the second step is to program the MAHAOS device by the same fixed voltage on control gate (refresh step) in each cycle. The upper curve is V*_T_* for the MAHAOS device under refresh step during each cycle and the lower curve represents the V*_T_* of the MAHAOS device in irradiation step during each cycle. As shown in the figure, the change of V*_T_* is not significant before 100 irradiation/refresh cycles, but the change of V*_T_* becomes more dramatically after 100 irradiation/refresh cycles. However, the radiation/refresh cycle characteristics of the MAHAOS device show better performance under low dosage of gamma irradiation conditions.

## 4. Conclusions

(1) As shown in the discussed experimental data, the negative shift of V*_T_* for the programmed MAHAOS device under gamma radiation is very significant. The radiation-induced shifts in the irradiated MAHAOS device are a combination of two effects; the first effect is a result from the loss of stored charge in the HfAlO trapping layer. The second effect is due to a build-up of positive charge resulting from asymmetric trapping of electrons and holes in the trapping layer. These two effects combined cause a large negative shift of V*_T_* in the pre-programmed MAHAOS under gamma radiation.
(2) As shown in the experimental results, the gamma irradiation causes a large decay of V*_T_* for the pre-programmed MAHAOS devices, while the gamma irradiation causes less shift of V*_T_* in the erased MAHAOS device. It is clearly observed in the experiment data that the effect of radiation on V*_T_* of the MAHAOS device depends on the initial magnitude of V*_T_* and hence the charge trapped in the HfAlO trapping layer prior to irradiation. So, the ionizing radiation effect causes a large negative V*_T_* shift on the pre- programmed and irradiated MAHAOS device.
(3) The data of the pre-programmed and irradiated MAHAOS device written by 5 Mrad TID of gamma irradiation was stable for a long time with data storage. For the high dose radiation sensing application, the gate leakage current is the key issue could be improved by using this new type MAHAOS device with high *k* stack gate dielectric. In summary, gamma radiation sensing by the pre- programmed MAHAOS devices using high *k* gate dielectric technology, which also is compatible with the high *k* CMOS processing technology, for gamma irradiation in this study has demonstrated promising potential to be used for ionizing radiation sensing application in the future.
